# Access to Neurosurgery for Patients in Germany—Strategic Considerations Based on Geographic Information Mapping

**DOI:** 10.3390/clinpract16020043

**Published:** 2026-02-20

**Authors:** Rosita Rupa, Anastasios Tsogkas, Dalibor Bockelmann, Christopher Nimsky, Benjamin Voellger

**Affiliations:** 1 Department of Neurosurgery, Marburg University Hospital, Baldinger Str., 35043 Marburg, Germany; rupar@med.uni-marburg.de (R.R.); nimsky@med.uni-marburg.de (C.N.); 2 Department of Neurosurgery, Hospital Kulmbach, Albert-Schweitzer-Str. 10, 95326 Kulmbach, Germany; anastasios.tsogkas@klinikum-kulmbach.de; 3 Staff Unit Medical Strategy and Cooperations, Medical Center, University of Freiburg, Breisacher Str. 153, 79110 Freiburg, Germany; dalibor.bockelmann@uniklinik-freiburg.de; 4 Department of Neurosurgery, St. Vincenz Hospital Paderborn, Am Busdorf 2, 33098 Paderborn, Germany

**Keywords:** geographic information mapping, German healthcare reform, hospital accessibility, neurosurgery, R

## Abstract

**Background/Objectives**: To estimate, against the background of the upcoming German healthcare reform, current access to neurosurgery for patients in Germany, and to derive improvement strategies from geographic information mapping. **Methods**: We defined access to neurosurgery on a geographical basis as the sum of all points from which one can reach a neurosurgical department within 40 min by car (A2N40). We identified 182 departments of neurosurgery, and we retrieved population numbers and geodetic information from open sources. We processed data and conducted statistical analyses in R. **Results**: Population density and A2N40 per square kilometer were significantly positively correlated (Spearman’s rho = 0.82, *p* = 0.0001). Population density is significantly lower (Wilcoxon rank sum test, *p* = 0.009) and A2N40 per square kilometer is significantly worse (Wilcoxon rank sum test, *p* = 0.005) in the new federal states (without Berlin) as compared to the rest of the country. Geographic information mapping yielded 3 distinct improvement strategies. **Conclusions**: In Germany, population density and A2N40 per square kilometer are significantly positively correlated, with significantly less A2N40 per square kilometer in the new federal states. Geographic mapping may inform tailored regional improvement policies.

## 1. Introduction

The upcoming reform of the German healthcare system [1] intends to integrate the demands of an ageing population [2], increased shortage of skilled workforce [3], and financial constraints. The reform assigns to each hospital a level of service and a set of medical specialties [1]. One basic requirement of the reform is that a considerable proportion of citizens can reach a hospital that provides certain medical specialties, namely general internal medicine and general surgery, within 30 min [1]. For the remaining medical specialties, such as neurosurgery, access within 40 min is considered adequate [1].

With the help of geographic information mapping, we aim to assess the current situation for neurosurgical patients in Germany from a healthcare system management perspective. We identified three key questions: (1) Where in Germany does access to a neurosurgical department within 40 min (A2N40) actually exist? (2) Are there differences in A2N40 between federal states? (3) How to improve the current situation?

## 2. Materials and Methods

We retrieved hospitals officially designated to run a neurosurgical department in Germany from federal hospital plans as of 20 Jun 2025 [4,5,6,7,8,9,10,11,12,13,14,15,16,17,18,19]. While not yet listed in a federal hospital plan, we decided to add Berufsgenossenschaftliche (BG) Klinik Ludwigshafen to our table of providers (Supplementary Table S1), as it has been announced that an officially designated neurosurgical department is about to be established there [20]. In total, we identified 182 departments of neurosurgery (Supplementary Table S1). We tabulated longitudes and latitudes of hospitals (i.e., coordinates of the respective emergency room driveway or, in case an approximation was needed, of the respective helicopter site) with the help of Google Maps [21] (Supplementary Table S1). In line with the upcoming reform of the German healthcare system, we defined access to neurosurgery on a geographical basis: we considered access to neurosurgery to be available where one can reach a neurosurgical department within 40 min by car. We obtained isochrones (i.e., polygonal outlines of areas of equal travelling time from/to a predefined point) for hospital coordinates from Openrouteservice [22] between June 2025 and December 2025 using R 4.4.1 [23] and R Studio 2024.09.1+394 [24] on a Mac OS X 12.7.6 [25]. We downloaded administrative-level geodetic information (i.e., polygonal outlines of Germany, its federal states and districts) from Geodatenzentrum des Bundesamts für Kartographie und Geodäsie (GDZ BKG) [26]. As required, we converted geodetic information to the World Geodetic System 1984 (WGS 84) and European Terrestrial Reference System 1989 (ETRS89) formats using R and R Studio. We queried absolute population numbers of German administrative districts as of 31 Dec 2023 at the Indikatoren und Karten zur Raum- und Stadtentwicklung (INKAR) [27] website of Bundesinstitut für Bau-, Stadt- und Raumforschung (BBSR). We retrieved population numbers and areas (in square kilometers) for Germany and its federal states from the website of the Statistisches Bundesamt (DESTATIS) [28].

We conducted statistics with R and R Studio. Due to the small number of 16 federal states, we cannot assume a normal distribution of population density at the federal state level (Kolmogorov–Smirnov test, *p* = 0.017). We therefore decided to apply rank sum tests. We considered the results of the Wilcoxon rank-sum test and the calculation of Spearman’s rank correlation coefficient (rho) significant at *p*-values < 0.05. We created figures with R, R Studio, a modified version of the R Shiny application introduced by authors B.V. and D.B. in 2024 [29], and the GNU Image Manipulation Program (GIMP) 2.10.34 [30].

## 3. Results

**Figure 1:**
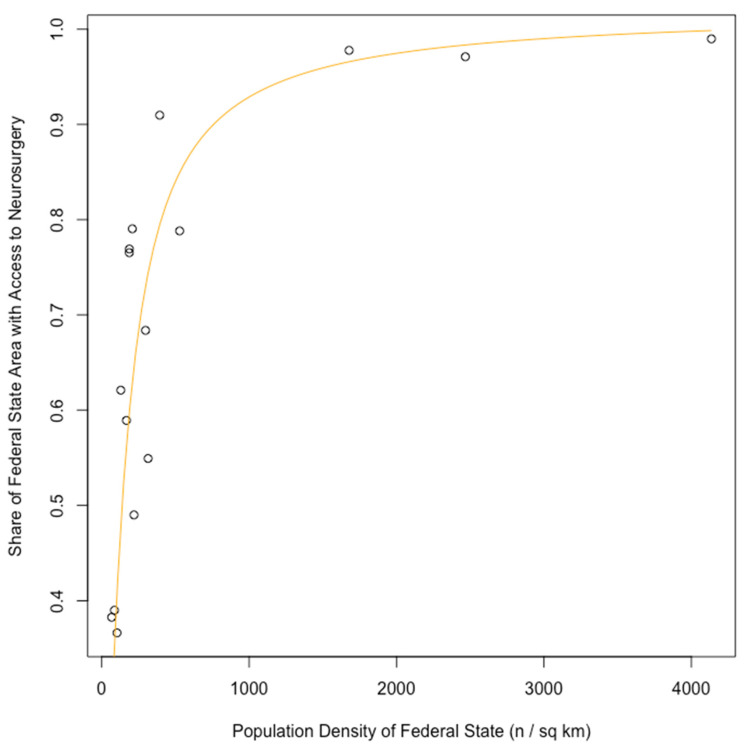
At the administrative level of German federal states (circles), there is a significantly positive correlation of population density with access to neurosurgery (i.e., the sum of all points from where a neurosurgical department can be reached within 40 min by car (A2N40)) per square kilometer (Table 1, Spearman’s rho = 0.82, *p* = 0.0001). The correlation follows a saturation curve (orange), which was manually fitted for the purpose of illustration according to the formula: y = (13/20) × arctangent (0.007 × x). Abbreviation: sq km—square kilometers.

At the administrative level of German federal states, population density and A2N40 per square kilometer are significantly positively correlated (Spearman’s rank correlation coefficient, rho = 0.82, *p* = 0.0001, Table 1, Figure 1).

**Figure 2:**
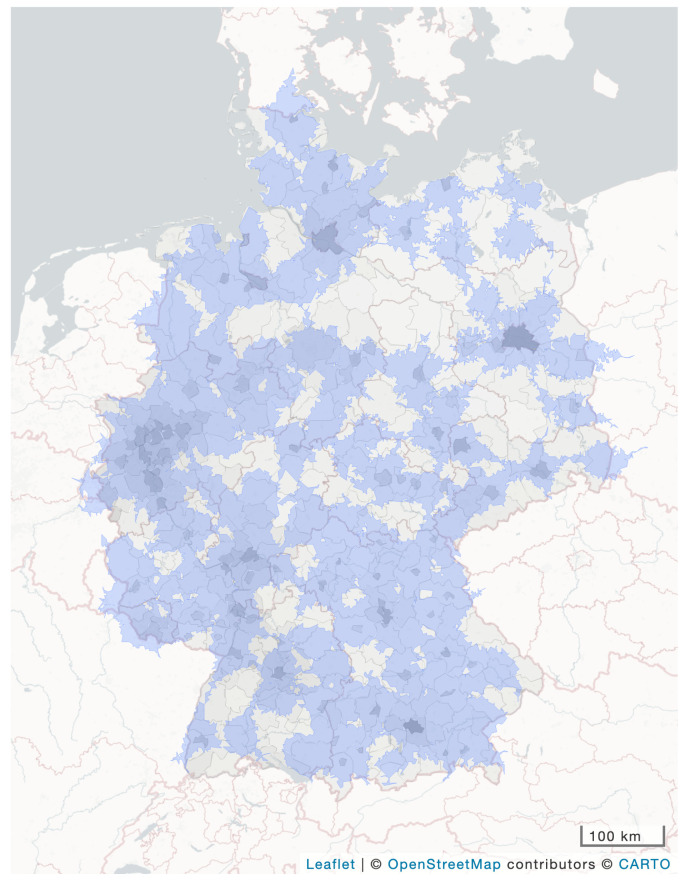
Population density per district (ground layer grey scale saturation increases with population density) and access to neurosurgery within 40 min by car (A2N40, blue overlay) in Germany.

Population density is significantly lower (Wilcoxon rank sum test, *p* = 0.009, Table 1, Figure 2) and A2N40 per square kilometer is significantly worse (Wilcoxon rank sum test, *p* = 0.005, Table 1, Figure 2) in the new federal states (without Berlin) as compared to the remaining territory of the country.

**Figure 3:**
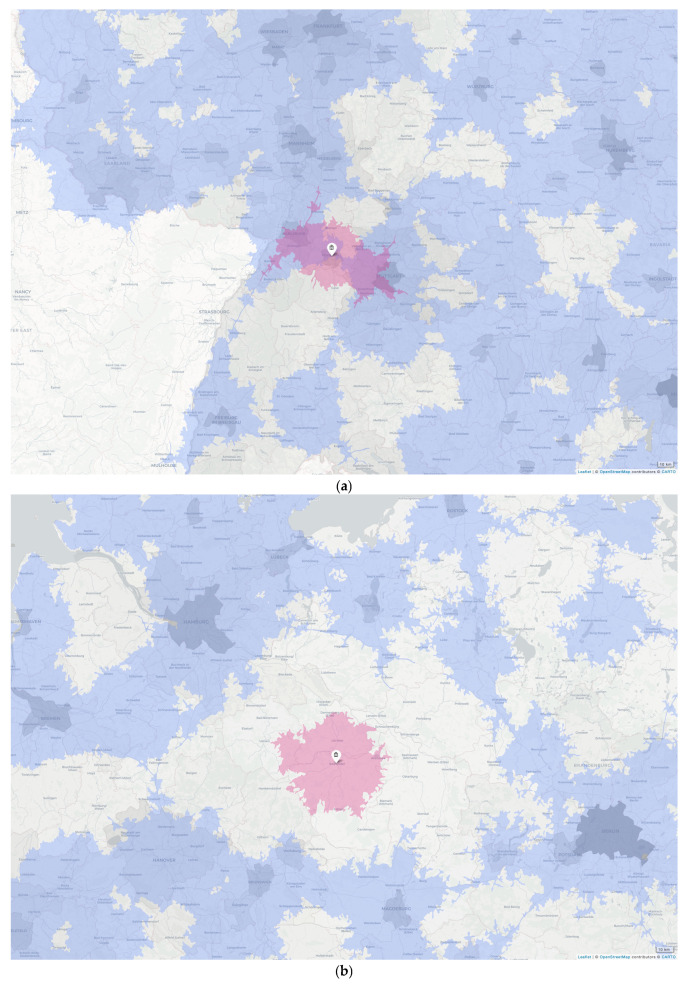
Potential increase (pink overlay) of current access to neurosurgery in Germany within 40 min by car (A2N40, blue overlay) if neurosurgical departments (pins) were established in (**a**) Pforzheim, Baden-Württemberg; (**b**) Salzwedel, Saxony-Anhalt in addition to the nearest neurosurgical departments in (clockwise, beginning in the north) (**a**) Mannheim, Heidelberg, Ludwigsburg, Stuttgart, Reutlingen, Tübingen, Karlsruhe, and Ludwigshafen; (**b**) Schwerin, Plau am See, Neuruppin, Berlin, Potsdam, Brandenburg, Magdeburg, Brunswick, Hanover, Rotenburg (Wümme), and Hamburg. Ground-layer grey scale saturation represents population density.

A closer examination of Figure 2 yields the following improvement strategies: Strategy A places a neurosurgical department where high population density meets low coverage (Figure 3a). Strategy B places a neurosurgical department, regardless of population density, where there is no coverage at all (Figure 3b)—strategies A and B increase A2N40. Strategy C leaves the distribution of neurosurgical departments as it is, while the mode and vector of transportation may be changed in an emergency (e.g., to fly the patient to the neurosurgeon, or vice versa).

## 4. Discussion

While it appears reasonable to establish a neurosurgical department in a region where high population density meets incomplete coverage (strategy A), addressing the absence of A2N40 in a less populated area demands careful consideration [31]. On the one hand, there is little or no A2N40 in the new federal states outside larger agglomerations. On the other hand, it is well known that the outcome of a surgical procedure depends on the surgeon’s experience, the availability and training of staff, the quality of equipment and facilities, and the caseload [32,33,34]. The aim to provide A2N40 for as many citizens as possible must therefore be balanced against the high degree of specialization and the expenditure for complementary medical specialties, which some neurosurgical procedures require.

One solution may be to allow a subset of neurosurgical procedures to be performed at hospitals in less populated regions. Procedures that require a higher degree of specialization may still require referral to an appropriate center.

Regarding access to neurosurgery for patients with spontaneous subarachnoid hemorrhage (SAH), Dinc et al. [31] have recently shown that 97 per cent of German citizens can reach a neurovascular center within 60 min where aneurysm obliteration is performed. This information indicates that there is sufficient coverage with neurosurgery services within a 60 min car drive (A2N60) in Germany.

As to the mode of transportation in emergencies (ground versus helicopter-based emergency medical services (HEMS)), a recently published retrospective analysis [35] in a large patient cohort (*n* = 887) of the German trauma register database found no significant differences in mortality, even after adjustment for potential bias. We agree with Ernstberger et al. [35] that choosing the most suitable hospital and means of transportation within a trauma network will ensure comparable outcomes for all trauma patients.

With any strategy, limitations may arise from a shortage of funding and staff. Scheduled rotations and other incentives may convince medical professionals to treat a limited range of diseases at a rather remote location. Skillful communication between all stakeholders is key to achieving the intended benefits in such a setting [34].

Notably, the German healthcare reform bill [1] defines time limits for access to medical specialties, while leaving room for interpretation regarding the number of beneficiaries in the population. For access to neurosurgical departments, the bill sets a 40 min time limit. Accordingly, we based our study on 40 min car-travelling isochrones. We found a significant correlation between the geographical coverage of neurosurgery services with population density at the level of federal states, and we mapped population density by district to identify strategies for improvement. However, our approach may not always adequately reflect local peculiarities.

We recommend always tailoring strategies to regional conditions. Occasionally, the optimal approach may involve a blend of strategies.

We froze information available from federal hospital plans on 20 June 2025. At that time, it had already been announced that a neurosurgical department was about to be established in Ludwigshafen. We therefore decided to add BG Klinik Ludwigshafen to our list of providers, and to consider the respective isochrone as an area with A2N40. However, we do not necessarily know all the political decisions regarding the designation of future neurosurgery providers in Germany.

It took us several months to collect all the data underlying this work. Meanwhile, road repair sites have changed. Modernization of a hospital may transform the emergency room driveway or the helicopter landing site. These factors may affect the calculation and currency of isochrones. We retrieved official population numbers as of 31 Dec 2023. While small changes in population numbers may have occurred afterward, they are unlikely to significantly affect results at the federal-state level.

While changes over time in isochrones and in population numbers are easy to understand, we found it interesting that the calculation of administrative level area based on geodetic information from different, albeit official, sources sometimes returns slightly different values (with changes in the range of about 1 per cent, or less). Data converted from one coordinate reference system to another appears to be particularly prone to this effect. We attribute this mainly to the R command “st_make_valid”, which we sometimes had to use in order to “repair” corrupted polygons.

This did not change the ranks of federal state population numbers, area, and A2N40 as listed in Table 1. We applied rank sum tests, so there is no impact on the statistical significance of our results. The small differences in area calculation, which we observed, certainly do not compromise the strategies we devise. However, our approach has limitations when highly accurate geographic information is required.

We mapped car-travelling isochrones primarily to derive improvement strategies on a qualitative level. Hence, our work does neither constitute a quantitative optimization nor is it meant for direct clinical application. The assessment of the current situation in other medical specialties in Germany was not the objective of this study. This work does not aim to investigate the potential benefits of cross-border cooperation. In order to estimate access to neurosurgery in other countries, different geographical or statistical approaches may be preferable, e.g., Dodds et al. [36] suggested elliptical isochrones for modeling HEMS in large rural areas of Scotland.

Despite all limitations, our study represents a systematic approach to analyzing current coverage with neurosurgery services in Germany. We demonstrate how geographic mapping of freely available information may help to find an appropriate strategy for regional improvements.

## 5. Conclusions

In Germany, population density and A2N40 per square kilometer are significantly positively correlated. Population density is significantly lower, and A2N40 per square kilometer is significantly worse in the new federal states as compared to the rest of the country. Geographic mapping may inform tailored regional improvement policies.

## Figures and Tables

**Table 1:** Germany and its federal states: population, area, and access to neurosurgery.

Administrative Unit *	Population	Area (km^2^)	A2N40 ** (Per Cent of Area)
Germany	83,577,140	357,684	62.03
Baden-Württemberg	11,245,898	35,748	54.91
Bavaria	13,248,928	70,542	76.91
Berlin	3,685,265	891	98.99
Brandenburg ^#^	2,556,747	29,654	39.00
Bremen	704,881	420	97.78
Hamburg	1,862,565	755	97.11
Hesse	6,280,793	21,116	68.37
Lower Saxony	8,004,489	47,710	58.91
Mecklenburg-West Pomerania ^#^	1,573,597	23,295	38.27
North Rhine-Westphalia	18,034,454	34,113	78.81
Rhineland-Palatinate	4,129,569	19,858	79.04
Saarland	1,012,141	2,572	90.97
Saxony ^#^	4,042,422	18,450	48.99
Saxony-Anhalt ^#^	2,135,597	20,555	36.64
Schleswig-Holstein	2,959,517	15,804	76.53
Thuringia ^#^	2,100,277	16,202	62.09

* Federal states in alphabetical order; ** A2N40 defined as: the area from where a hospital with a neurosurgical department can be reached within 40 min by car (equal to the blue layers in Figure 2 and Figure 3); ^#^ new federal state.

## Data Availability

All underlying data and the R code used to visualize the results of this study are freely available from open sources.

## Supplementary Materials

The following supporting information can be downloaded at: https://www.mdpi.com/article/10.3390/clinpract16020043/s1, Table S1: list of hospitals with longitudes and latitudes.

## Abbreviations

The following abbreviations are used in this manuscript:

A2N40	Access to Neurosurgery within 40 min
A2N60	Access to Neurosurgery within 60 min
BBSR	Bundesinstitut für Bau-, Stadt- und Raumforschung
BG	Berufsgenossenschaft(liche)
DESTATIS	Statistisches Bundesamt
ETRS89	European Terrestrial Reference System 1989
GDZ BKG	Geodatenzentrum des Bundesamts für Kartographie und Geodäsie
GIMP	GNU Image Manipulation Program
HEMS	Helicopter-based Emergency Medical Service
INKAR	Indikatoren und Karten zur Raum- und Stadtentwicklung
OS	Operating System
SAH	Subarachnoid Hemorrhage
sq km	Square Kilometers
WGS 84	World Geodetic System 1984
